# Intra-arterial ethanol embolization augments response to TACE for treatment of HCC with portal venous tumor thrombus

**DOI:** 10.1186/s12885-018-3989-2

**Published:** 2018-01-29

**Authors:** Biao Yang, Chun-Lin Li, Wen-hao Guo, Tian-qiang Qin, He Jiao, Ze-jun Fei, Xuan Zhou, Lin-jia Duan, Zheng-yin Liao

**Affiliations:** 1 Department of Abdominal Oncology, Cancer Center and State Key Laboratory of Biotherapy, West China Hospital, West China Medical School, Sichuan University, Guoxue Lane No. 37, Chengdu, Sichuan Province 610041 People’s Republic of China; 2 Chinese Evidence-Based Medicine Centre, West China Hospital, West China Medical School, Sichuan University, Chengdu, People’s Republic of China; 3 Department of Radiology, West China Hospital, West China Medical School, Sichuan University, Chengdu, People’s Republic of China

**Keywords:** Portal vein tumor thrombus, Transcatheter arterial chemoembolization, Hepatocellular carcinoma, Cone-beam computed tomography

## Abstract

**Background:**

The prognosis of hepatocellular carcinoma with portal vein tumor thrombus remains extremely poor. This pilot study aimed to evaluate the technical feasibility, effectiveness and safety of transcatheter chemoembolization for tumors in the liver parenchyma plus intra-arterial ethanol embolization for portal vein tumor thrombus.

**Methods:**

A pilot study was carried out on 31 patients in the treatment group (transcatheter chemoembolization plus intra-arterial ethanol embolization) and 57 patients in the control group (transcatheter chemoembolization alone). Enhanced computed tomography/magnetic resonance images were repeated 4 weeks after the procedure to assess the response. Overall survival and complications were assessed until the patient died or was lost to follow-up.

**Results:**

Median survival was 10.5 months in the treatment group (2.4 ± 1.7 courses) and 3.9 months in the control group (1.9 ± 1 courses) (*P* = 0.001). Patients in the treatment group had better overall survival (at 3, 6 and 12 months, respectively), compared to patients in the control group (90.3% vs. 59.6%, 64.5% vs. 29.8%, and 41.9% vs. 10.6%; *p* = 0.001). Furthermore, the rate of portal vein tumor thrombus regression was higher in the treatment group (93.1%) than in the control group (32.1%) (*P* < 0.001).

**Conclusions:**

Based on the results of this study, transcatheter chemoembolization combined with intra-arterial ethanol embolization may be more effective than transcatheter chemoembolization alone for treating hepatocellular carcinoma with portal vein tumor thrombus. Intra-arterial ethanol embolization for treating portal vein tumor thrombus is safe, feasible and prolongs overall survival.

## Background

Hepatocellular carcinoma (HCC) is the fifth most frequently diagnosed cancer worldwide and the third most frequent cause of cancer death [1, 2]. Unfortunately, HCC has a propensity to invade the portal vein and cause portal vein tumor thrombus (PVTT) [3], and this can be detected in 30-62% of patients with HCC [4]. PVTT is considered as an adverse prognosis factor [3]. Although liver resection and liver transplantation are accepted as the only potential curative treatment for HCC patients, HCC with PVTT has been considered a contraindication to surgery due to poor prognosis and high surgical risk [5]. Both percutaneous ethanol injection (PEI) and radiofrequency ablation have not been shown to improve survival in cases of HCC with neoplastic involvement of major branches of the portal vein or main portal trunk (Vp3/Vp4), and median survival ranged from 2.4 to 4.8 months [6]. For patients with PVTT, sorafenib is suggested as the standard therapy of care in the Barcelona Clinic Liver Cancer (BCLC) staging system [7, 8]. However, the median overall survival (OS) gain with sorafenib is 5.6 months, and better treatment modalities are clearly required [9]. Yamada et al. [10] performed TACE in nine patients with PVTT (Vp4), and 1-month mortality was 55.5%. Among those patients, 33% of patients died of hepatic insufficiency. Based on this study, they concluded that TACE was contraindicated in HCC patients with PVTT (Vp4). Recently, two studies have indicated that transarterial chemoembolization (TACE) could be safely performed in such patients with no increase in morbidity or mortality [11, 12]. Most importantly, all methods described in these studies are targeting intrahepatic lesions, and none of these focused on treating PVTT itself. In addition to treating intrahepatic lesions, it is our hypothesis that a therapeutic approach including the treatment of the portal vein thrombus itself could provide benefits in terms of OS.

Ethanol can produce an embolization effect by causing endothelial damage and thrombus of the arteriolar lumen of tumor feeder vessels and tumor vasculature, thereby leading to tumor infarction [13]. Intra-arterial lipiodol-ethanol mixture embolization has been shown to be effective for treating HCC [14, 15]. Si et al. [16] revealed that the feeding vessels of PVTT are complex. However, in their study, 92.3% of PVTT had the same blood supply characteristics as intrahepatic lesions, indicating that most nutrient vessels of PVTT correspond to liver arteries. C-arm cone beam computed tomography (CACT) angiography could be helpful to identify the PVTT-feeding artery and embolize the PVTT by lipiodol-ethanol mixture. In addition, CACT provides a good method for evaluating iodized oil deposition during the procedure. Based on these data, we considered that intraarterial ethanol embolization for PVTT could be feasible and effective. In the present study, we present a new lipiodol-ethanol mixture technique, wherein, intraarterial ethanol embolization and TACE are combined to treat HCC patients with PVTT (Vp3/Vp4).

## Methods

### Study design

**Fig. 1:**
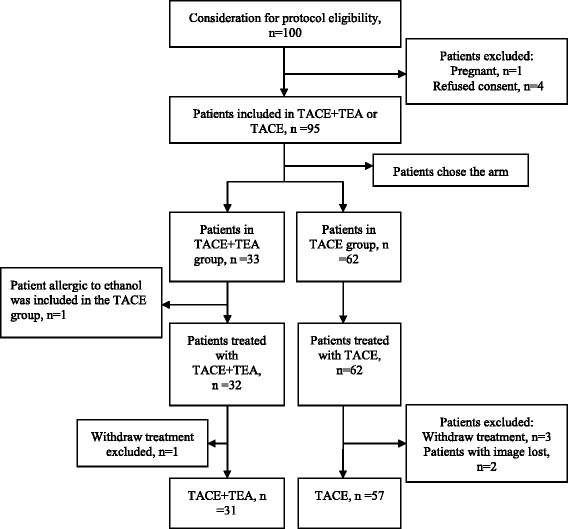
Study flow chart

This cohort study was approved by the Local Ethics Committee of West China Hospital, Sichuan University. A written informed consent was obtained from each patient after being informed of the purpose and investigational nature of the present study. This study was conducted according to the Declaration of Helsinki, and strictly adhered to the CONSORT guidelines. Participants were recruited from June 2014 to November 2016. Patients were stratified into two groups according to their willingness (Fig. 1). These patients were followed up until the date of analysis in January 2017. Among these patients, 31 patients received TACE plus intraarterial ethanol embolization (treatment group), while 57 patients received TACE only (control group).

### Eligibility criteria

All these patients were preoperatively evaluated by abdominal ultrasonography and thoracoabdominal dynamic computer tomography (CT)/magnetic resonance imaging (MRI), while some patients underwent abdominal angiography. The extent of the tumor thrombus to the portal vein was accurately assessed through these imaging techniques.

#### Inclusion criteria

(1) patients with unresectable HCC with PVTT (Vp3/Vp4); (2) patients with no history of any disease-specific treatment including surgery in the past 6 months; (3) patients who had both an international normalized ratio of < 1.5 and Child-Pugh class A/B cirrhosis; (4) patients with an Eastern Cooperative Oncology Group (ECOG) score ≤ 2; (5) patients who provided an informed consent; (6) patients who had no serious concurrent medical illness; (7) patients with histologically or cytologically proven HCC, except for lesions > 2 cm, with typical features of one dynamic imaging technique and α-fetoprotein level > 40 ng/mL; (8) patients who had a tumor size of up to 18 cm in the largest dimension; (9) patients with treatment failure with sorafenib or those who refused to receive sorafenib as treatment for advanced HCC; (10) patients who were allergic to ethanol and refused to be included in the treatment group, and were thereby included in the control group.

#### Exclusion criteria

(1) patients who were ≥ 75 years old or < 18 years old; (2) females who were pregnant; (3) patients who had a history of variceal bleeding within the past 3 months; (4) patients with active hepatitis B (HBV-DNA > 1000 copies/ml); (5) patients who have a history of acute tumor rupture with hemoperitoneum; (6) patients with concurrent ischemic heart disease or heart failure; (7) patients with a history of hepatic encephalopathy; (8) patients with thrombosis of the target hepatic artery.

## Procedure

### Intra-arterial ethanol embolization for PVTT

All TACE and intra-arterial ethanol embolization procedures were performed by two operators using the same angiographic system (Allura Xper FD20, Philips Healthcare). Prophylactic antibiotic treatment was not given. The treatment procedure was performed under local anesthesia with 3–5 mL of 1% lidocaine (Lidocaine Hydrochloride Injection, Taiji, Chongqing, China) administered at the groin, and started with hepatic arteriography to identify the tumor. Intra-arterial ethanol embolization procedures were performed using a 2-F tip microcatheter (Progreat α; Terumo Clinical Supply, Gifu, Japan) through a 4F catheter, in order to identify the potential PVTT-feeding artery under digital subtraction angiography (DSA). Furthermore, an angiographic unit with a 38 × 30 cm^2^ flat panel detector (FPD) was used to obtain CACT images and confirm the PVTT-feeding artery. For each CACT scan, 312 projection images with X-ray parameters of 120 kV and 200-300 mAs were acquired with the motorized C-arm, covering a 200° clockwise arc at a rotation speed of 20° per second. Then, 6-20 mL of contrast material (370 mg I/mL; Omnipaque, Bracco-Sine, Shanghai, China) were injected under 100-300 MPa over 6-10 s with a 2-s delay. After confirmation of the PVTT-feeding artery and before delivery of the ethiodized oil–ethanol solution, 1 mL of 1% lidocaine was instilled intra-arterially through the microcatheter at each site of the solution administration for pain control. Lipiodol-ethanol mixture (1:1 ratio by volume up to 15 mL) was injected at a rate of 0.5-1 ml/min until the PVTT-feeding artery was nearly occluded, followed by embolization with 0.2-0.5-mm gelatin-sponge particles (Gelfoam; Fukangseng, Guilin, China) using a three-way stopcock valve and two 2.5-mL syringes. The agents were delivered under fluoroscopic control until the vasculature of all tumors was entirely filed, as shown by the fluoroscopic evidence of intraarterial flow stasis or until the maximum dose was reached. In case of acute severe abdominal pain, the procedure was temporarily suspended or stopped.

### Intra-arterial ethanol embolization in managing different types of PVTT

It was observed that repeating the treatment at 3-4 weeks after the first treatment was often necessary to achieve good results, since achieving complete intra-arterial ethanol embolization in a single session was difficult in most patients. Further treatment sessions were administered when there was CT evidence of residual tumors or occurrence of new hepatic tumors. There was no limit on the total number of treatment sessions.

### Simple type (PVTT with only one or two feeding arteries)

Intra-arterial embolization was performed in most patients to treat the PVTT-feeding artery. The lipiodol-ethanol mixture was slowly injected in the feeding-artery, followed by a gelatin sponge mixed with contrast material. If the patient experienced acute intense pain, further injection was stopped or delayed. Two advantages of using a gelatin sponge were observed. First, it avoids lipiodol-ethanol mixture regurgitation, and decreases the rate of cholecystitis and bile leakage. Second, it stays within the target tissue for a long time and at a higher concentration, accounting for better lipiodol deposition on post-procedure CT scan. Then, an epirubicin-lipiodol mixture was injected into the intrahepatic lesions through TACE with a mixture of lipiodol (10 ml) and epirubicin (50 mg; Pfizer, Wuxi, China), followed by a gelatin sponge mixed with contrast material, until the vasculature of all tumors was entirely filed, as shown by fluoroscopic evidence of intra-arterial flow stasis.

### Brush type

**Fig. 2:**
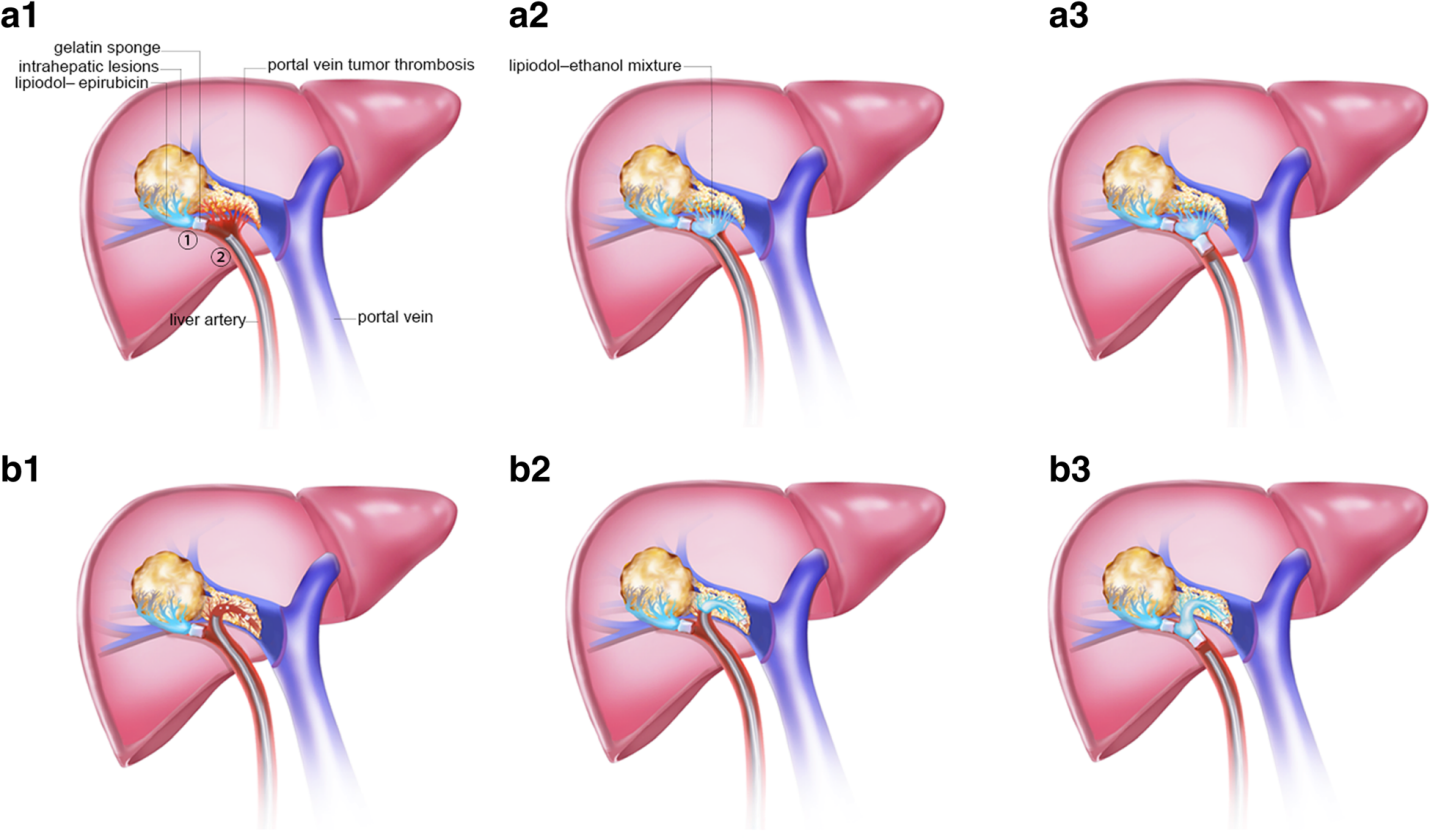
Intra-arterial ethanol embolization procedure for different types of PVTT. (A1) A microcatheter was inserted into place: (1) epirubicin injection followed by a gelatin sponge. (A2-A3) The microcatheter was withdrawn from its location: (2) lipiodol-ethanol mixture injection (1 ml/s), followed by a gelatin sponge. (B1) Same method as described in A1. (B2) A microcatheter was placed to permit the gelatin sponge to block the draining vessel. (B3) Lipiodol-ethanol mixture injection followed by gelatin sponge is shown

Some patients with PVTT had several small tortuous feeding-arteries (Fig. 2, A1-A3). Hence, it was difficult to directly insert the microcatheter into the feeding-artery due to anatomical variations in its location. In our technique, for this type of PVTT, TACE was first performed in intrahepatic lesions until stasis distal to small tortuous feeding arteries. Then, the lipiodol-ethanol mixture was injected in the nearby PVTT-feeding artery, followed by a gelatin sponge. Throught this method, high concentrations of lipiodol-ethanol mixture flowing through the PVTT-feeding artery could be achieved.

### Large arteriovenous fistula type

In patients with large arteriovenous fistulas, after measuring the diameter of the vessel, the distal outflow vessel was embolized using a larger diameter gelatin sponge before injecting the lipiodol-ethanol mixture (Fig. 2, B1-B3), avoiding the mixture from flowing out too fast. This helped to maintain the high lipiodol-ethanol mixture concentration in the feeding-artery for a longer time, providing enough time for diffusion to the PVTT.

### TACE for tumors in the liver parenchyma

TACE with a mixture of lipiodol and epirubicin (50 mg of epirubicin; Pfizer, Wuxi, China) was performed for intrahepatic lesions, followed by gelatin-sponge embolization under DSA without CACT scan. Before the catheter was removed from the artery, diluted heparin was injected (50 IU/ml, 10 ml).

### Follow-up and assessment indices

**Fig. 3:**
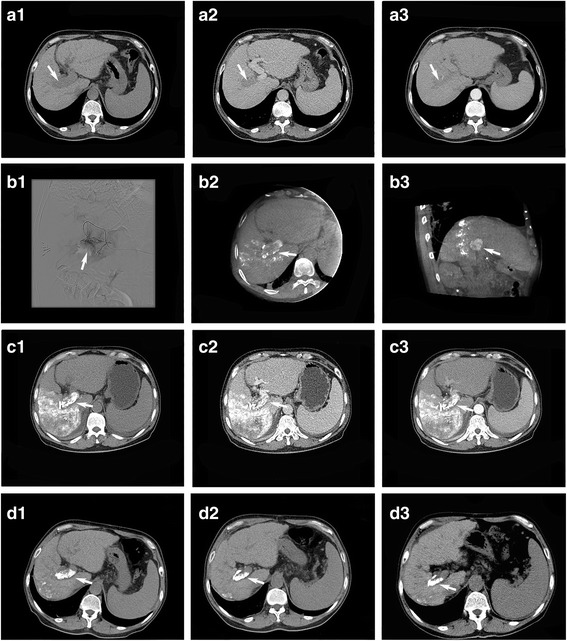
Intraarterial ethanol embolization with TACE in a 64-year-old male with HCC and PVTT (Vp3). (A1, A2) CT scan in the portal venous phase highlighting PVTT in the right portal vein (arrow) is shown; (A3) PVTT-feeding artery identified on CT. (B1) PVTT-feeding artery identified on DSA by superselective catheterization of the feeding artery using a microcatheter; (B2, B3) enhanced C-arm CT was performed to further confirm the PVTT-feeding artery; (C1-C3) axial CT showing lipiodol-ethanol mixture deposition within PVTT. (D1-D3) Follow-up images showing stable lipiodol-ethanol mixture deposition within PVTT at 3, 6, and 12 months after the operation

Primary outcomes were the overall response of PVTT to therapy and OS. Adverse events were considered as secondary outcomes. The latest version of the Response Evaluation Criteria In Solid Tumors (RECIST) guidelines (version 1.1) were used to assess the tumor response of intrahepatic lesions to therapy [17]. Considering that the tissue organization of postoperative residual thrombi without viability can be persistent for months or years, and that PVTT is always accompanied by benign thrombus [18], the investigators decided not to adopt the RECIST guidelines in assessing the efficiency on PVTT. Therefore, the following four grades were proposed to classify PVTT response to therapy: grade 3, recanalization of the portal vein trunk or, right or left portal vein; grade 2, decreased PVTT diameter without recanalization of any branch of the portal vein; grade 1, neither shrinkage to qualify for grade 2 nor increase to qualify for grade 0; grade 0, PVTT diameter increased by 20%. Regression of PVTT to grade3/grade2 and complete/partial response of intrahepatic lesions based on the modified RECIST criteria were considered as significant responses to interventional therapy. At 2-7 days after the procedure, CT scan revealed lipiodol deposits within PVTT (Fig. 3, C1-D1). Enhanced CT/MRI images, which were evaluated by two experienced radiologists, were repeated at 4 weeks after the procedure, in order to assess the response. OS was defined as the period from the date of first treatment to the date of death, or censorship at the date of last follow-up if the patient is still alive. Repeated TACE was performed if lesion diameter increased or new lesions were found. Repeated intra-arterial ethanol embolization was suspended if PVTT diameters did not increase, or complete embolization of the visible PVTT-feeding artery was achieved.

### Statistical analyses

Continuous baseline characteristic variables were compared by Student *t*-test, ranked data was compared by rank-sum test, and categorical variables were compared by *χ*^*2*^-test. Survival curves were estimated using the *Kaplan-Meier* method. Data were analyzed with SPSS version 20.0 (SPSS Inc., Chicago, IL, USA). All statistical tests used were two-sided, and *P* < 0.05 was considered statistically significant.

## Results

### Patient demographics

In the treatment group, the mean age of patients (*n* = 31) was 54.3 ± 11.9 years old. These patients received TACE with 50 mg of epirubicin dissolved in 10 ml of lipiodol for intrahepatic lesions combined with intra-arterial ethanol embolization (alcohol/lipiodol, 7.8 ± 3.9 ml) for PVTT (Fig. 3). In the control group, the mean age patients (*n* = 57) was 54.2 ± 13.4 years old. These patients received TACE with 50 mg epirubicin alone (Table 1). The mean course of procedures in the treatment and control groups was 2.4 ± 1.7 and 1.9 ± 1.0, respectively (*P* = 0.18). Results related to the end-points of the present study are summarized in Table 2.

**Table 1:** Baseline patient characteristics

Variable	Treatment group (*n* = 31)	Control group (*n* = 57)	*P*-value
Age^a^ (year)	54.29 ± 11.87	54.16 ± 13.42	0.96
Gender^b^			0.52
Male	25 (80.6)	49 (86)	
Female	6 (19.4)	8 (14)	
Classification of PVTT^b^			0.37
Vp3	10 (32.3)	24 (42.1)	
Vp4	21 (67.7)	33 (57.9)	
ECOG performance^b^			0.43
0	17 (54.8)	34 (59.6)	
1	9 (29.1)	20 (35.1)	
2	5(16.1)	3(5.3)	
Cause of liver disease^b^			1.00
HBV	31 (100)	57 (100)	
Other	0 (0)	0 (0)	
Liver Cirrhosis^b^			0.75
Absent	8 (25.8)	13 (22.8)	
Present	23 (74.2)	44 (77.2)	
Distant metastasis^b^			0.53
Lung	2 (6.5)	2 (3.5)	
Others	0 (0)	0 (0)	
Ascites^b^			0.71
Absent	26 (83.9)	46 (80.7)	
Mild	1(3.2)	3(5.3)	
Moderate	3(9.7)	7(12.3)	
Massive	1(3.2)	1(1.7)	
Child-Pugh score^b^			0.03
A	25 (80.6)	33 (57.9)	
B	6 (19.4)	24 (42.1)	
Tumor description^b^			0.83
Number of tumors			
1	6 (19.4)	11(19.3)	
2	4 (12.9)	9(15.8)	
≥ 3	21 (67.7)	37 (64.9)	
Size of largest tumor, median (cm)	7.00	8.5	0.34
Angiography of PVTT feeding-artery			
Time^a^ (seconds)	8.65 ± 1.74	–	
Pressure^a^ (MPa)	243.55 ± 92.86	–	
Speed^a^ (ml/s)	1.63 ± 0.43	–	
Procedure			
Total procedure Time^a^ (hour)	1.92 ± 0.62	0.62 ± 0.18	< 0.001
Alcohol^a^ (ml)	7.77 ± 3.95	–	
Epirubicin^a^ (mg)	50.00	50.00	
Number of sessions	2.42 ± 1.71	1.89 ± 0.98	0.18
Laboratory Tests			
Total bilirubin level^a^ (μmol/L)	22.15 ± 12.63	20.91 ± 9.19	0.60
Albumin level^a^ (g/L)	39.22 ± 8.12	41.68 ± 5.91	0.11
α-Fetoprotein^b^ (ng/mL)			0.34
< 200	10 (23.8)	22 (38.6)	
200–1000	4 (29)	11 (19.3)	
> 1000	17 (42.9)	24 (42.1)	
Prothrombin time^a^ (seconds)	12.86 ± 1.66	13.01 ± 1.17	0.42
Thrombin time^a^ (seconds)	19.41 ± 2.35	19.71 ± 1.23	0.63

^a^Mean ± standard deviation (SD); ^b^*n* (%)

**Table 2:** Comparison of primary and secondary outcomes between the treatment and control groups

Outcome	Treatment group	Control group	*P*-value
Tumor response^b^	*n* = 29	*n* = 53	0.61
Complete response	2 (7)	6 (11.3)	
Partial response	9 (31)	5 (9.4)	
Stable disease	12 (41.3)	35 (66.1)	
Progressive disease	6 (20.7)	7 (13.2)	
Portal vein tumor thrombus response^b^	*n* = 29	*n* = 53	< 0.001
Grade 3	6 (20.7)	1 (1.9)	
Grade 2	15 (51.7)	6 (11.3)	
Grade 1	6(20.7)	10 (18.9)	
Grade 0	2 (6.9)	36 (67.9)	
Overall survival (%)	*n* = 31	*n* = 57	0.001
At 3-months	90.3	59.6	
At 6-months	64.5	29.8	
At 12-months	41.9	10.6	

Values are depicted as *n* (%)

^b^Before the statistics were performed, two patients died in treatment group and control group, respectively

### Survival

**Fig. 4:**
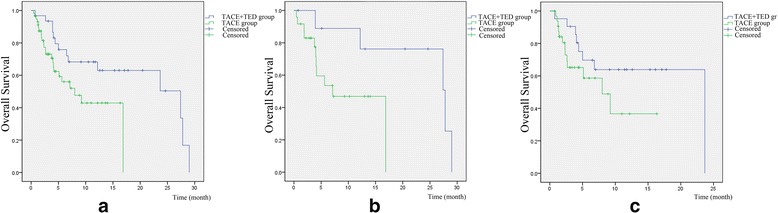
A graphical representation of the overall survival of patients in the two groups by the *Kaplan-Meier* method. **a** The total overall survival curve in the two groups. **b** The overall survival of patients diagnosed with Vp3 in the two groups. **c** The overall survival of patients with Vp4 in the two groups

Thirty-eight patients died during follow-up: 14 patients in the treatment group and 24 patients in the control group. Median survival was 10.5 months and mean survival was 11.5 ± 8.5 months (95% CI: 8.6-15.3) in the treatment group, while median survival was 3.8 months and mean survival was 5.0 ± 4.0 months (95% CI: 4-6) in the control group (Fig. 4). Probabilities of survival at 3, 6 and 12 months were significantly higher in the treatment group than in the control group (90.3% vs. 59.6%, 64.5% vs. 29.8%, and 41.9% vs. 10.6%) (*P* = 0.001, Table 2). The mean survival of patients classified as Vp3 and Vp4 in the treatment and control group was 16.4 ± 10.8 vs. 9.2 ± 6.1 (*P* = 0.004) and 5.9 ± 4.7 vs. 4.3 ± 3.7 (*P <* 0.001). The mean survival of patients classified as Child-Pugh A and Child-Pugh B in the treatment and control groups was 13.2 ± 12.6 vs. 4.0 ± 3.0 (*P <* 0.001) and 11.0 ± 9.4 vs. 5.2 ± 4.4 (*P* = 0.003), respectively.

### Safety

Ninety complications occurred in the treatment group, while 125 complications occurred in the control group. The duration of the procedure was significantly longer in the treatment group than in the control group (1.9 ± 0.6 h vs. 0.6 ± 0.2 h, respectively; *P* < 0.001). No treatment-related death, pulmonary embolism, renal damage, renal failure, respiratory failure, cholecystitis, or cholangitis were observed during follow-up in both groups. The other main adverse events are presented in Table 3.

**Table 3:** Comparison of adverse events related to the procedure

Adverse event	Treatment group (*n* = 31)	Control group (*n* = 57)
Fatigue	16	18
Gastrointestinal hemorrhage	2	1
Fever	14	19
Abdominal pain	31	56
Vomiting	8	15
Chest pain	2	0
Per procedure vomiting	3	1
Back pain	12	7
Loss of appetite	2	8
Total	90	125

### Significant clinical response

Complete response/partial response/stable/progressive disease of intrahepatic lesions at 1 month was observed in 7%, 31%, 41.3% and 20.7% of patients in the treatment group, compared to 11.3%, 9.4%, 66.1%, and 13.2% of patients in the control group, respectively (*P* = 0.61, Table 2). PVTT radiographic response rate to therapy was significantly higher in the treatment group (37.9%), compared with the control group (13.2%) (*P* < 0.001, Table 2).

## Discussion

PVTT is an independent prognostic factor for patients with HCC. The reported median survival for untreated HCC patients with PVTT (Vp3/Vp4) was 2.7 months, whereas survival in patients without PVTT was 24.4 months [3]. Intra-arterial ethanol embolization has been used in HCC cases in a similar approach to TACE, which has exhibited a higher 1-year OS rate (93.3% vs. 73.3%) and greater lipiodol retention (89.5% vs. 47.5%); but its specific impact on PVTT has not been previously studied [19]. The present study demonstrated that our therapeutic approach may be more effective than TACE in HCC patients with PVTT (Vp3/Vp4). The percentage of patients with more than three nodes or diffused tumors, or huge tumors in the treatment group was higher than in the control group. This selection bias may be due to the patient’s decision, and may have lessened the possibility to evaluate the advantages in the treatment group. Despite this limitation, there was a significant trend toward OS improvement vs. the control group. We found that occluding the arterial supply to PVTT with the help of intraarterial ethanol embolization not only resulted in the recanalization of the portal vein, but also significantly improved survival in this patient group. In the treatment group, patients had more complications compared with those in the control group, which was possibly correlated to the destruction of the PVTT-feeding artery induced by ethanol. The potential risk of thrombus at the infusion port was higher due to longer procedure time in the treatment group. This is why some blood was extracted from both the infusion port and connected catheter before removing the needle from the infusion port, followed by flushing with diluted heparin. The duration of post-procedure abdominal pain was longer in the treatment group than in the control group, which was possibly due to the ethanol itself.

Yamada et al. [10] first reported that HCC patients with Vp3/Vp4 PVTT treated with TACE had a 28.6% 1-year survival rate. Recently, Chung et al. [20] reported a 30% 1-year survival rate and 28.2% PVTT response rate. Georgiades et al. [12] and Takayasu et al. [21] reported a 25% and 35% 1-year survival rate, respectively. Peng et al. [11] reported that PVTT had a 36.1% 1-year survival rate. In contrast, in the present study, the 1-year survival rate was 41.9% for patients in the treatment group vs. 10.6% for patients in the control group, which show that patients in the treatment group had a higher OS rate than those reported in literature [11, 12]. In the present study, PVTT response rate was higher than those were reported by Yamada et al. [10]. Percutaneous ethanol injection has been reported to be efficient for treating PVTT on the basis that ethanol is diffused within cells [22]. Nevertheless, ethanol in PEI is limited both in terms of diffusion and of adapting the ethanol dose. In contrast, intra-arterial ethanol embolization, a method based on the infusion of ethanol into the artery, can achieve a more efficient diffusion without damaging the normal liver parenchyma, and allows the ethanol dose to be easier controlled according to the pain degree of the patient or the distribution of ethanol.

Diagnosing PVTT remains difficult. Percutaneous puncture biopsy is invasive, and is associated with a high risk of tumor seeding along the needle track. Color Doppler sonography (CDS) has been widely used with the method of pulsatile flow. PVTT has been diagnosed in approximately 62% of patients using the presence of a pulsatile flow as its diagnostic criterion [23]. DSA together with CACT combines the advantages of DSA and contrast-enhanced CT, which can provide slice imaging and dynamic flow information. Wallace et al. [24] reported that 60% of CACT images contain information that were not found in DSA, and influenced the treatment procedure in 19% of cases. CACT detects HCC with greater accuracy and sensitivity than both DSA and CT [25]. In addition, CACT data sets can be viewed in three-dimension and slice-images. These information provides a more effective therapy by delivering an increased amount of lipiodol-ethanol mixture to the target, while sparing uninvolved parenchyma exposure from toxic agents such as the gallbladder.

In an animal experiment carried out by Kan et al. [13] and the use of absolute ethanol, the endothelial cell was denuded from the vascular wall, its protoplasm precipitated and a fracture in the vascular wall to the level of the internal elastic lamina was formed, followed by the shrinking of lesions. Hence, ethanol has been widely used for vascular malformations [16]. Ethanol is a better embolic agent than lipiodol, and can lead to vascular endothelial destruction. However, ethanol is not radio-opaque, and its flow and speed are difficult to visualize. In contrast, lipiodol-ethanol mixture (in a 1:1 ratio) is visible during injection. At the same time, it maintains the potency of absolute ethanol in the target vasculature, and is not diluted by aqueous solutions; which are necessary to avoid regurgitation and ectopic embolization [15]. From the fluoroscopic observation on an animal model, dual embolization could be induced by the slow infusion of an insoluble substance such as the lipiodol-ethanol mixture, which appears as small droplets passing through the hepatic sinusoids and to the portal vein [14]. This achieves complete embolization in both arteries that supply the tumor and its adjacent parenchymal portal veins [14]. The long-lasting embolization of both the arterioles and portal venules is highly effective in causing infarction of the whole tumor including the tumor border, which is commonly supplied by portal venules [26]. The treatment group, unlike a gelatin sponge, not only induces tumor ischemia and hypoxia, but also diffuses within tumor cells [15, 27, 28]. Ischemia and hypoxia may be potent stimulators of angiogenesis and carcinogenesis, which promote collateral circulation and the restoration of tumor blood supply; and these may eventually lead to tumor proliferation and recurrence [29, 30].

### Limitations

The main limitations of the present study are small sample size, non-randomized controls, relatively short follow-up, and a single center experience. Therefore, performing prospective randomized studies are warranted to confirm these results. Any new treatment should ideally be compared with the reference standard for the disease at that stage. The evidence based standard of care for locally advanced HCC is sorafenib. However, few Chinese people able to bear the high cost, especially in developing countries [31]. Moreover, there is no standard treatment for patients with treatment failure with sorafenib. A recent study [32] demonstrated that patients with PVTT (Vp3), who received TACE or sorafenib, had a poor 1-year OS (35.7 vs. 26.5 months). Hence, for this group of patients, we propose TACE treatment. Although, there was no statistical significance between the compared groups in terms of treatment courses, more number of courses of repeated TACE in HCC provided better results in the treatment group. Clearly, a higher proportion of Child-Pugh A patients in the treatment group may contribute to explain the longer OS.

## Conclusion

Although intra-arterial ethanol embolization combined with TACE does not represent a cure for HCC with PVTT, the principal goals of significant safety, effectiveness and OS could be achieved. In the present pilot study, considering the higher survival rate for TACE plus intra-arterial ethanol embolization compared with TACE alone, this therapeutic approach may be the treatment of choice for HCC patients with PVTT (Vp3/Vp4). However further prospective studies are needed to confirm the present data.

## Abbreviations

CACT: C-arm cone beam computed tomography
CDS: Color doppler sonography
CT: Computer tomography
DSA: Digital subtraction angiography
ECOG: Eastern Cooperative Oncology Group
FPD: Flat panel detector
HCC: Hepatocellular carcinoma
MRI: Magnetic resonance imaging
OS: Overall survival
PEI: Percutaneous ethanol injection
PVTT: Portal vein tumor thrombus
RECIST: Response evaluation criteria in solid tumors
TACE: Transarterial chemoembolization
